# Atlantic Leatherback Migratory Paths and Temporary Residence Areas

**DOI:** 10.1371/journal.pone.0013908

**Published:** 2010-11-09

**Authors:** Sabrina Fossette, Charlotte Girard, Milagros López-Mendilaharsu, Philip Miller, Andrés Domingo, Daniel Evans, Laurent Kelle, Virginie Plot, Laura Prosdocimi, Sebastian Verhage, Philippe Gaspar, Jean-Yves Georges

**Affiliations:** 1 Département Ecologie, Physiologie et Ethologie, Université de Strasbourg, IPHC, Strasbourg, France; 2 CNRS, UMR7178, Strasbourg, France; 3 Satellite Oceanography Division, Collecte Localisation Satellites, Ramonville St Agne, France; 4 Departamento de Ecologia, Universidade do Estado do Rio de Janeiro, Rio de Janeiro, Brazil; 5 Karumbé, Villa Dolores Zoo, Montevideo, Uruguay; 6 Centro de Investigación y Conservación Marina, El Pinar, Canelones, Uruguay; 7 Dirección Nacional de Recursos Acuáticos, Montevideo, Uruguay; 8 Sea Turtle Conservancy, Gainesville, Florida, United States of America; 9 WWF Guianas, Cayenne, French Guiana; 10 Regional Program for Sea Turtles Research and Conservation of Argentina, PRICTMA, Buenos Aires, Argentina; 11 WWF Gabon, Libreville, Gabon; Lund University, Sweden

## Abstract

**Background:**

Sea turtles are long-distance migrants with considerable behavioural plasticity in terms of migratory patterns, habitat use and foraging sites within and among populations. However, for the most widely migrating turtle, the leatherback turtle *Dermochelys coriacea*, studies combining data from individuals of different populations are uncommon. Such studies are however critical to better understand intra- and inter-population variability and take it into account in the implementation of conservation strategies of this critically endangered species. Here, we investigated the movements and diving behaviour of 16 Atlantic leatherback turtles from three different nesting sites and one foraging site during their post-breeding migration to assess the potential determinants of intra- and inter-population variability in migratory patterns.

**Methodology/Principal Findings:**

Using satellite-derived behavioural and oceanographic data, we show that turtles used Temporary Residence Areas (TRAs) distributed all around the Atlantic Ocean: 9 in the neritic domain and 13 in the oceanic domain. These TRAs did not share a common oceanographic determinant but on the contrary were associated with mesoscale surface oceanographic features of different types (i.e., altimetric features and/or surface chlorophyll *a* concentration). Conversely, turtles exhibited relatively similar horizontal and vertical behaviours when in TRAs (i.e., slow swimming velocity/sinuous path/shallow dives) suggesting foraging activity in these productive regions. Migratory paths and TRAs distribution showed interesting similarities with the trajectories of passive satellite-tracked drifters, suggesting that the general dispersion pattern of adults from the nesting sites may reflect the extent of passive dispersion initially experienced by hatchlings.

**Conclusions/Significance:**

Intra- and inter-population behavioural variability may therefore be linked with initial hatchling drift scenarios and be highly influenced by environmental conditions. This high degree of behavioural plasticity in Atlantic leatherback turtles makes species-targeted conservation strategies challenging and stresses the need for a larger dataset (>100 individuals) for providing general recommendations in terms of conservation.

## Introduction

Many species show considerable behavioural plasticity in terms of foraging and habitat use in response to fluctuations in environmental conditions and prey availability [1]–[5], or to changes in energetic requirements associated with the different stages of the annual cycle (e.g., reproduction, migration [6]–[8]). In addition, a high degree of phenotypic plasticity usually exists between geographically separate populations experiencing different ecological conditions. For instance, rockhopper penguins *Eudyptes chrysocome* from three different colonies in the Indian Ocean have been reported to show significant differences in diving behaviour and foraging effort with consequences on life history traits such as chick growth [9]. Similarly, gravid green turtles *Chelonia mydas* have been shown to exhibit contrasted, probably food-mediated, patterns of depth utilisation between Ascension Island (mid-Atlantic) and northern Cyprus (Mediterranean Sea) [10].

High degree of behavioural plasticity within a species may make species-targeted conservation strategies more difficult to implement. For instance, Cape gannets *Morus capensis* from two colonies off South African coasts show contrasted foraging strategies: birds from one colony feed on natural prey, i.e. pelagic fish targeted by fisheries, while occupants of the second colony feed mainly on fishery wastes [11]. Therefore some fisheries may increase food availability for gannets through waste while other fisheries compete directly with the birds when harvesting their main natural prey, making the implementation of any conservation policies in this area particularly challenging [12]–[14]. This example highlights the difficulty of implementing efficient conservation strategies at a species level without taking into account inter-population variability in terms of foraging and dispersal behaviour.

Sea turtles are long-distance migrants that exhibit a high variability in migration destination among individuals of a same population and among populations [15]. The potential determinants of migration destination have recently been investigated in the loggerhead turtle *Caretta Caretta* from a major rookery in the Mediterranean [16]. It appeared that the pattern of adult dispersion from the breeding area closely matched the different drift scenarios that would have been experienced by hatchlings as they first left their natal beach. In their early lives as they passively drift in ocean currents, turtles may explore different habitats and potential future foraging areas. Then, as adults, they may use this initial experience to migrate to predictable foraging sites. This hypothesis of “hatchling drift scenarios” has also been suggested to explain the genetic connectivity between geographically distant populations of green turtles [17].

The critically endangered leatherback turtle, *Dermochelys coriacea*, performs the longest migration of any sea turtle and disperses throughout all the ocean basins (e.g., [18]–[22]) to forage on patchily-distributed jellyfish [23]. Many studies have investigated in details the diving, foraging and dispersal behaviour of leatherback turtles and show a high degree of variability among individuals of the same population [19]–[21], [24]–[35]. Yet, to date only one study described the spatio-temporal foraging patterns of satellite-tracked leatherback turtles from different nesting and foraging sites in the North Atlantic [27]: this study showed a similar degree of behavioural variability among individuals and among populations in Atlantic leatherback turtles.

Here, we investigated the movements and diving behaviour of both north and south Atlantic leatherback turtles during the post-breeding migration of 12 individuals from three different nesting sites and 4 individuals captured at one foraging site to assess the potential determinants of intra- and inter-population variability in migratory patterns. We particularly focused on oceanographic conditions encountered by the turtles during the migration in order to test potential hatchling drift scenarios at the Atlantic Ocean scale.

## Methods

### Ethics statement

This study adhered to the legal requirements of the countries in which the work was carried out, and to all institutional guidelines. Fieldwork in French Guiana and Suriname was carried out under CNRS-IPHC institutional license (B67 482 18) and individual licences to JYG (67-220 and 04-199) and SF (67-256) delivered by the National Committee of Nature Protection (French Ministry of Ecology and Sustainable Management), Paris, France; the Departmental Direction of the Veterinary Services, Strasbourg, France; and the Police Prefectures of Bas-Rhin and French Guiana. In Uruguay the fieldwork was conducted by Karumbe under a permit of scientific capture and collection (# 73/08) from the Fauna Department - Ministry of Cattle, Agriculture and Fishing. In Gabon, fieldwork was conducted by WWF Gabon which has an “accord de siege” (i.e. “headquarter agreement”) from the Ministère des Eaux et Forêts of Gabon and who has been recognized to do fieldwork on marine turtles in this area since 2002. In Panama, fieldwork was conducted by Caribbean Conservation Corporation under the permits SE/A-55-04 and SE/A-48-05 delivered by the Autoridad Nacional del Ambiente (ANAM).

### Turtles and satellite tracking

Sixteen satellite transmitters (Series 9000 Satellite Relayed Data Loggers SRDLs, manufactured by the Sea Mammal Research Unit, St. Andrews, United Kingdom) were deployed on leatherback turtles between June 2005 and October 2006 (**Table 1**) within the Trans-Atlantic Leatherback Conservation Initiative (TALCIN, see acknowledgements). Three tags were deployed on the Caribbean coast of Panama at Chiriqui beach (9.0°N-81.7°W), one in Suriname at Samsambo beach (5.8°N-54.0°W), five in French Guiana at Awala-Yalimapo beach (5.7°N-53.9°W) and three in Gabon at Kinguere beach (0.2°N-9.2°W). One turtle was equipped in Uruguay at Kiyu (34.7°S-56.7°W) after it was incidentally captured by an artisanal bottom-set gillnet, and three were equipped in international waters of the Southwestern Atlantic (29.5°S-41.7°W; 28.3°S-44.0°W and 28.2°S-44.3°W respectively) after they were incidentally captured by Uruguayan pelagic longliners. Among these 16 turtles, 14 were mature females, one was a mature male (UR06-2) and one a subadult (UR06-1; **Table 1**). Most of the tagged animals were females as, for logistical reasons, fieldwork mainly occurred at the nesting sites. Some of these tracks have been previously published [20], [26], [36] but not the post-breeding migrations of the turtles nesting in Gabon, which are described for the first time in the present study. For all turtles, SRDLs were attached on the pseudo-carapace using custom-fitted harness systems except for two turtles (FG05-4 and FG05-5) for which SRDLs were directly attached to the carapace [36].

**Table 1:** Summary of the movements of 16 Argos tracked leatherback turtles during their migration between 2005 and 2008.

Turtle	Deployment location	SCCL (cm)	Sex	Date of departure	Track duration (days)	Minimum travelled distance (km)
FG05-1	French Guiana	147	F	26 Jul 2005	164	6048
FG05-2	French Guiana	160	F	26 Jul 2005	410	9971
FG05-3	French Guiana	-	F	28 Jul 2005	258	7048
FG05-4	French Guiana	-	F	27 Jul 2005	103	5212
FG05-5	French Guiana	149	F	25 Jul 2005	113	6005
SU05-1	Surinam	148	F	25 Jun 2005	715	14154
PA05-2	Panama	152	F	13 Jun 2005	632	17614
PA05-4	Panama	152	F	08 Jul 2005	362	9200
PA05-5	Panama	156	F	16 Jun 2005	324	11289
GA06-1	Gabon	160	F	04 Mar 2006	533	11096
GA06-2	Gabon	163	F	05 Mar 2006	109	2834
GA06-3	Gabon	143	F	05 Mar 2006	299	6120
UR05-1	International waters	148	F	15 Jun 2005	314	8184
UR06-1	International waters	126	unknown	14 Aug 2006	340	6636
UR06-2	International waters	159	M	31 Jul 2006	237	5957
UR06-3	Uruguay	156	F	29 Oct 2006	631	15362

### Turtle movement analysis

Turtle movements were reconstructed using the Argos satellite location system (www.cls.fr). Inter-nesting tracks occurring during the nesting season were not included in the analysis. All tracks were processed in a similar way as in Gaspar et al. [37]: all locations of all accuracies were analysed, however Argos locations implying an apparent speed above 2.8 m.s^−1^ (i.e. >10 km.h^−1^) were discarded as travel rates above this threshold are considered as biologically unlikely [32]. Tracks were then smoothed and re-sampled every 3 hours. This sampling interval provides a spatial resolution sufficient for sampling the mesoscale variations of the ocean current fields and thus correctly estimating the currents along the tracks (see below). A local linear regression with a time window of two days was used to re-sample the tracks. Epanechnikov kernel was used to weigh observations in that window, and eventually adjust the size of the window according to the quality of the data in order to avoid over-smoothing the tracks. Re-sampled tracks (hereafter referred as apparent path) were analysed in three ways, as described below.

First, thanks to the regular re-sampling interval used, we calculated the time spent in 1° latitude by 1° longitude areas along the apparent paths in order to distinguish sections where turtles spend significantly more or less time, hereafter referred as Temporary Residence Areas (TRAs) and transit areas, respectively. When considering the cumulative frequency distribution of the time spent per 1° * 1° area, the curve reveals an inflection at the y-point corresponding to 90 hours (i.e. 76.1%). Accordingly, we considered that for each turtle, a TRA could be defined as 1° * 1° area where the animal spent at least 90 h. All tracks were thus divided into several sections (TRA *vs* transit) for which behavioural parameters were calculated (see below).

Secondly, due to the impact that ocean currents may have on an animals' movements [37]–[39] we estimated the surface currents experienced by each individual in order to distinguish the animal's apparent path (including a current drift component) from its own swimming motion (hereafter referred as motor path). In short, this consisted of computing surface velocity fields on a daily basis, by summing the geostrophic and Ekman components deduced from altimetry and wind stress data, respectively (www.aviso.oceanobs.com). Then, at each 3-h re-sampled location, we calculated (1) an apparent velocity, (2) a local surface current velocity and (3) a swimming velocity, corresponding to the difference between the apparent and the current velocities. This current correction was performed for all turtles except those remaining at low latitudes (<10°) where geostrophic approximations break down [37].

Last, we considered that an animal could stay in any given TRA either by decreasing its travel rate or by modifying the spatial structure of its apparent path, i.e. its apparent path straightness. Straightness variations can be detected along a path by successively measuring the ratio *D*/*L* for path sections with a constant length *L*. Consistently, each apparent path was re-sampled in a form of a sequence of *n* steps with a constant length *l* (*l* = 15 km in the present study, corresponding to the average distance between our successive Argos locations), and the ratio *D*_i_/*L* was successively calculated for each location (x_i_, y_i_) at the centre of a 10-steps (*L* = 150 km) window, i.e. between location (x_i-5_, y_i-5_) and location (x_i+5_, y_i+5_). To further investigate the relation between the apparent path and the swimming behaviour of the turtle, the same procedure was applied to the motor paths.

### Turtle diving behaviour

SRDLs provided measurements of diving behaviour from a pressure sensor, which sampled depth every 4 seconds with an accuracy of 0.33 m. Data were statistically summarised onboard over 6-h collection periods providing the number of individual shallow (between 2 and 10 m) and deep (>10 m) dives performed during the period, their mean (± SD) duration and mean (± SD) maximum depth, as well as the proportion of time spent at the surface and diving (in shallow or deep waters). SRDLs continuously logged summaries but only a sample of these data was relayed by satellite because of the limited bandwidth of the Argos link. For each temporary residence/transit area identified as above, the above mentioned dive parameters were averaged for statistical analyses.

### Satellite-derived oceanographic data

In addition to the estimation of the surface current fields (see above), the oceanographic regions crossed by the turtles were characterised using bathymetry, chlorophyll *a* data and altimetry. Bathymetry data were issued from the National Geophysical Data Center, National Oceanic and Atmospheric Administration, U.S. Department of Commerce, at a spatial resolution of 1/30° (ETOPO2v2; www.ngdc.noaa.gov). The seafloor regimes were subdivided as follows: neritic (i.e. continental shelf waters (<200 m) and shelf slope (200 to 2000 m)) and oceanic (>2000 m). Chlorophyll *a* surface concentration was described using monthly grids produced by the SeaWiFS project (spatial resolution of 9 km; http://web.science.oregonstate.edu/ocean.productivity/). Altimetry data obtained from AVISO (www.aviso.oceanobs.com) provided weekly maps of sea level anomaly (MSLA) and maps of absolute dynamic topography (MADT) on a 1/3 * 1/3° Mercator grid. Both MSLA and MADT data underwent a time linear interpolation to obtain daily gridded fields.

### Drifter data

To assess the potential drift scenarios of passive particles from our different tagging sites, we used the Global Lagrangian Drifter Data (http://www.aoml.noaa.gov/envids/). This dataset consists of satellite-tracked buoys drogued near the surface (15 m) from 1979 to the present. Drifter locations are estimated from 16 to 20 satellite fixes per day, per drifter. The Drifter Data Assembly.

Center (DAC) at NOAA's Atlantic Oceanographic and Meteorological Laboratory (AOML) assembles these raw data, applies quality control procedures and interpolates them via kriging to regular 6-h intervals. Here we selected satellite-tracked buoys that have passed within a window with ±5° of amplitude in longitude and latitude (1) centred on each tagging site or (2) centred on a particular TRA.

## Results

### Migration patterns

**Figure 1:**
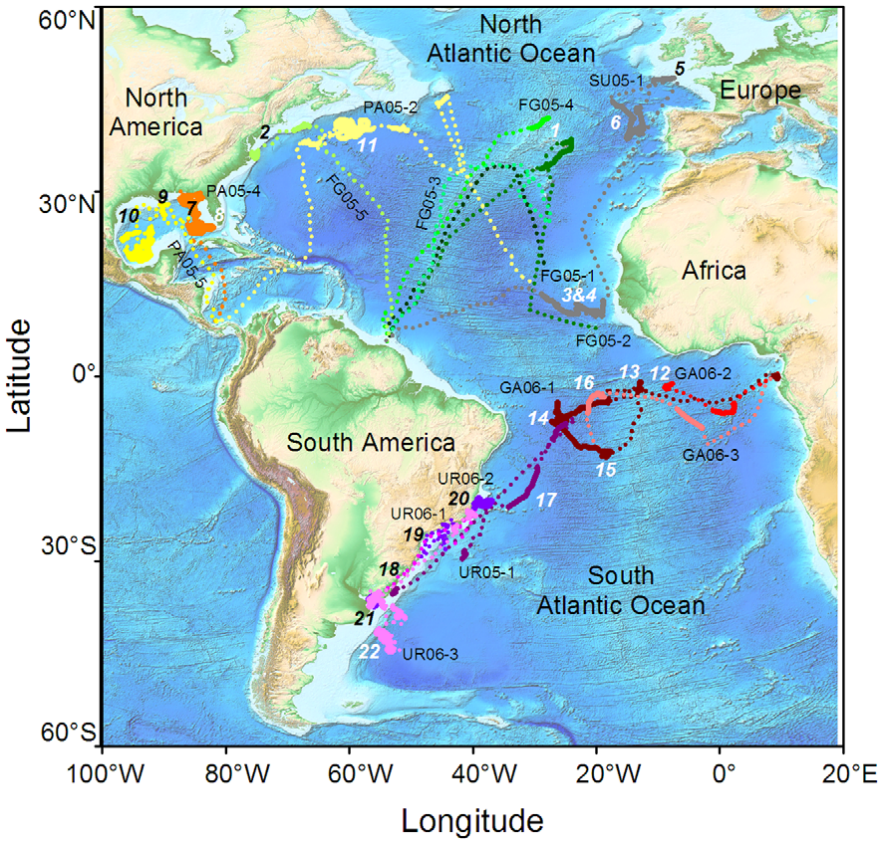
Movements of 16 leatherback turtles. Reconstructed movements of 16 Argos-tracked leatherback turtles during their migration in the Atlantic Ocean from 2005 to 2008. Twelve SRDLs were deployed on gravid females nesting in Panama (n = 3, PAyear-ID), Suriname and French Guiana complex (n = 6, SUyear-ID and FGyear-ID, respectively), and Gabon (n = 3, GAyear-ID). Four others were deployed on leatherback turtles incidentally captured by Uruguayan fisheries (pelagic longlines and coastal bottom-set gillnets) in international waters of the Southwest Atlantic and in Kiyú, Uruguay, respectively (URyear-ID). For each turtle, transit and Temporary Residence Areas (TRAs) are identified by dotted and solid lines, respectively. Each TRA is identified by a number in black and white, for neritic and oceanic domains, respectively (see M&M for details).

Tracking duration of the sixteen turtles ranged from 103 days (FG05-4) to 715 days (SU05-1) for recorded distances ranging from 2834 to 17 614 km (**Table 1**). Distinct dispersal patterns were observed according to the tagging location and 22 Temporary Residence Areas (TRAs) were identified (**Fig. 1**).

#### Suriname - French Guiana complex

The six females which left French Guiana and Suriname between June and July 2005 dispersed widely but remained into the North Atlantic. Four females dispersed north-eastward (FG05-1, FG05-2, FG05-3 and FG05-4), reaching the Azores Front (between 34°N and 41°N, TRA1) at the end of summer/beginning of autumn. They spent between several weeks to several months in this oceanic area before three of them headed south at the end of autumn/beginning of winter towards the Cape Verde islands. One female headed north-westward (FG05-5) and reached the Eastern continental shelf of USA (TRA2) in October 2005 where she remained until transmission stopped one month later. The last female (SU05-1) dispersed eastward reaching the Guinea Dome area (between 10°N -14°N and 23°W -19°W, TRA3) in October 2005. She stayed in this oceanic area until March 2006 before reaching the Mauritania upwelling area (TRA4) where she remained for two months. In May, she travelled north to the Bay of Biscay (TRA5) where she spent one month. In November, she moved south and spent the next six months until June 2007 off the coasts of Portugal (TRA6).

#### Panama

Two out of the three turtles equipped in Panama in July 2005 and June 2006 dispersed in the Gulf of Mexico while the third one reached the North Atlantic. After crossing the Caribbean Sea in one month, one turtle (PA05-4) explored the eastern side of the Gulf of Mexico spending two months (Sep-Oct 2005) along the north-eastern continental slope (TRA7) and four months (Nov 2005-Mar 2006) south of the Loop Current (TRA8). The second turtle (PA05-5) first moved towards the Northern continental shelf of the Gulf of Mexico (TRA9) and then travelled to the Western and South-western shelves of the Gulf (TRA10) from August to September 2006 towards an area between Vera Cruz and Yucatan (Mexico) where she remained during six months until March 2007. The third turtle (PA05-2) reached the Gulf Stream in October 2005 after crossing the Caribbean Sea. She remained in this oceanic area (between 36°N-42°N and 69°W-50°W, TRA11) during five months, before migrating southeast by March 2006 towards the Cape Verde Islands.

#### Gabon

**Figure 2:**
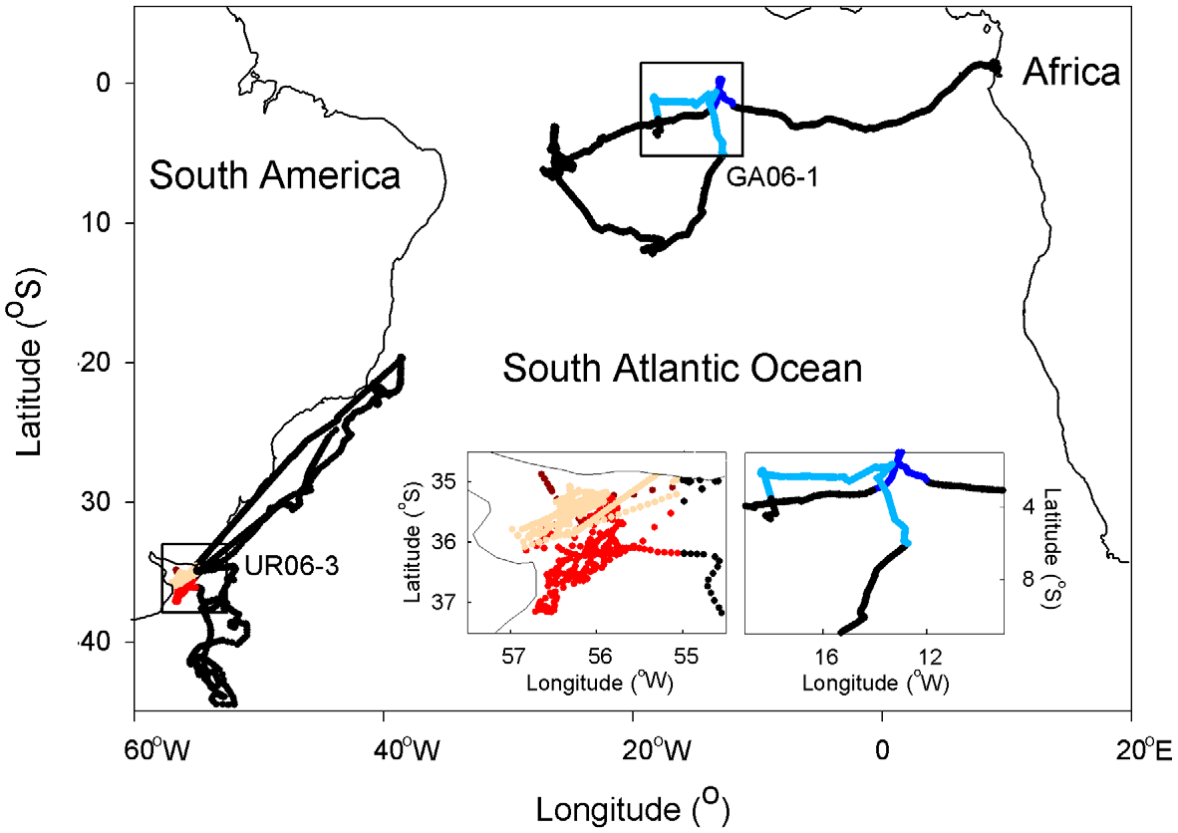
Fidelity to Temporary Residence Areas. Illustrative examples of fidelity to Temporary Residence Areas (TRAs) in leatherback turtles during their pluri-annual migration. After nesting in Gabon in March 2006, GA06-1 reached a first oceanic TRA (TRA13) by May 2006 (right insert, dark blue track) that she reached again by May 2007 (light blue track) after a counter-clockwise long loop in the middle South-equatorial Atlantic. After being released in the Rio de la Plata estuary in October 2006 (left insert, brown track), UR06-3 moved southward into oceanic water before coming back to her neritic TRA: the Rio de la Plata estuary (TRA21) by February 2007 (red track) that she reached again by January 2008 (orange track) after migrating north toward Brazilian waters close to the Victoria-Trinidad seamount chain. Each year, UR06-3 resided during 3 months in the Rio de la Plata estuary (TRA21).

The three turtles which left Gabon in March 2006 (GA06-1, GA06-2 and GA06-3), dispersed in the South Atlantic and remained within the South Equatorial Current between 0° and 13°S. Tracking of turtle GA06-2 ended in June 2006 while she was still in the Gulf of Guinea at 1°S–8°W (TRA12). GA06-1 reached a first oceanic area (1°S–13°W, TRA13) by May 2006 (**Fig. 2**) where she remained during one month before moving westward to another oceanic area located between 8°S–4°S and 27°W–25°W (TRA14) where she spent three months (Aug-Nov 2006) before reaching a last oceanic area situated at 12°S–18°W (TRA15) where she remained two months (Jan-Feb 2007). She then returned north-eastward approximately to the same oceanic area where she was in June 2006 (TRA13) and spent one month there before transmission ceased. Turtle GA06-3 spent four months (Jul-Oct 2006) close to the equator (1°-4°S, TRA16), then moved to the same oceanic area where turtle GA06-1 (TRA15) was located between January and March 2007, just before transmission ceased.

#### Uruguay

All four turtles which were released after being incidentally captured in the open ocean off the Uruguayan coast (n = 3) and in coastal waters of the Rio de la Plata (n = 1) in June 2005, August and October 2006 dispersed within the South-western Atlantic. The turtle UR05-1 moved north-eastward, slowed down around 20°S–30°W (TRA17) and reached 6°S–24°W at the end of November 2005 where GA06-1 also remained between August and November 2006 (TRA14). After one month in this oceanic area, she moved back towards the Uruguayan continental shelf (TRA18) where she was last located in April 2006. The sub-adult UR06-1 remained in the Southern Brazilian Bight (between 23°S and 29°S, TRA19) during its entire tracking. The male UR06-2 first moved north-eastward until 21°S and spent September between the continental slope and the Victoria-Trinidad seamounts (TRA20). He then travelled back along the continental shelf and reached the Rio de la Plata estuary (TRA21) in November 2006 where he remained until transmission stopped in March 2007. The turtle UR06-3 left the Uruguayan continental shelf in November 2006 and reached the Brazil-Malvinas Confluence area (TRA22) where she remained for two months (Dec 2006-Jan 2007). She came back to the Rio de la Plata estuary (TRA21) in early March 2007 where she stayed for three months (**Fig. 2**). Then she moved north-eastward along the Uruguayan and Brazilian continental shelves. From August 2007 to September 2007, she remained close to the Victoria-Trinidad seamounts and the continental slope (TRA20). She returned to the Rio de La Plata (TRA21) in January 2008 (**Fig. 2**). After spending >4 months in the estuary, she headed northeast towards tropical waters before transmissions ceased in July 2008.

### Drifter trajectories

**Figure 3:**
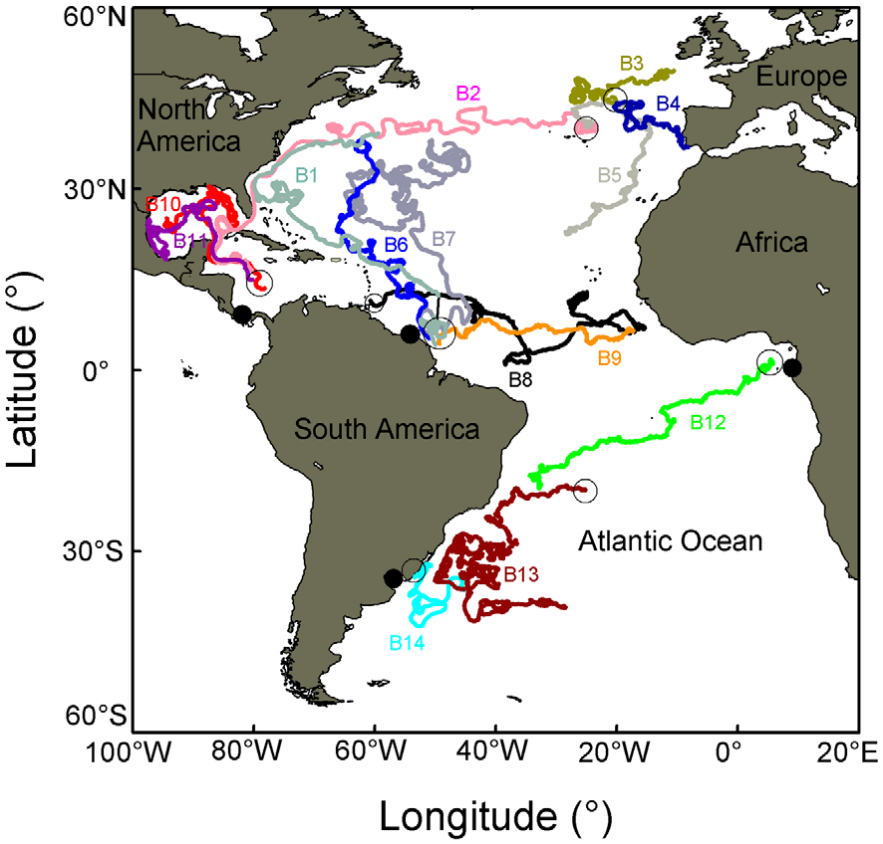
Trajectories for satellite-tracked drifters. Map of trajectories for satellite-tracked drifters released in the vicinity of leatherback turtle tagging sites. Filled circles show the location of the tagging sites. Dotted circles show the starting point of the drifter tracks. Drifters were selected to indicate possible drift scenarios from the tagging sites (Panama, Suriname, French Guiana, Gabon and International waters off the Uruguayan coasts) to the main Temporary Residence Areas of the leatherback turtles identified in this study.

Buoys travelling off the French Guiana-Suriname coasts have been shown to drift in different directions (**Fig. 3**). First, northwest towards the North American coasts (B1) and then possibly drift into the Gulf Stream until they reach the Azores (B2). From the Azores, the buoys can travel northward to the Irish Sea and the Bay of Biscay (B3), eastward to the Iberian coasts (B4), or southward to the Cape Verde islands, *via* the Canaries Islands (B5). Secondly, buoys can travel broadly northward to the Gulf Stream area (B6 and B7) and then drift to the east (B2). Last, they can travel eastward to the African coasts reaching the Guinea Dome area (B8 and B9). Buoys travelling off the Panama coasts (**Fig. 3**) can travel first northward to the Gulf of Mexico, and then possibly disperse either to the east (B10) or to the west into the Gulf (B11) or travel eastward by drifting into the Gulf Stream (B2). Buoys travelling off the Gabon coasts (**Fig. 3**) can travel westward into the South Atlantic Gyre (B12), from where they can end up on the South American continental shelf (B13), they can then travel south-eastward along the Brazilian coasts (B13). Buoys travelling off the Uruguay coasts (**Fig. 3**) can travel southward to the Brazil-Malvinas confluence area (B14). Although such data should be taken with caution as they were collected at different periods, they suggest that passive objects may drift from our different tagging sites and reach all the leatherback TRAs identified in this study, in approximately 1 to 3 years.

### Environmental characteristics of temporary residence areas

**Figure 4:**
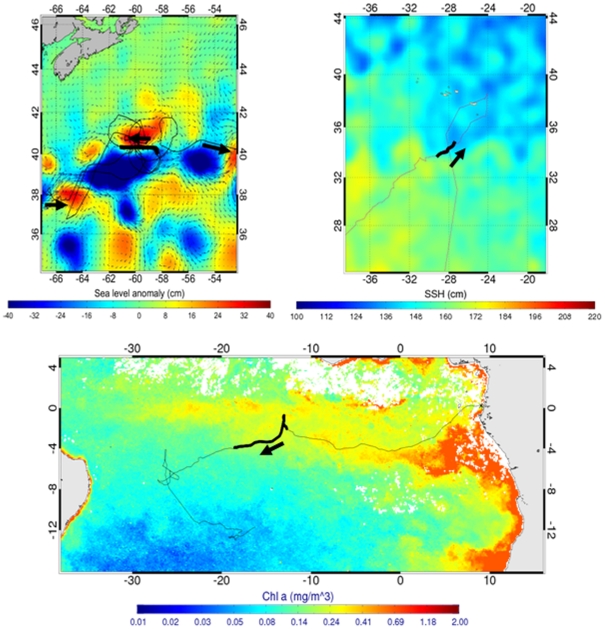
Migration paths and oceanographic parameters. a- Migration path in relation to weekly sea level anomaly (MSLA) of an Argos-tracked leatherback turtle (PA05-2) nesting in Panama in July 2005. The fine line represents the turtle's track from 10/10/2005 to 20/02/2006 (TRA11), while the bold line represents the week from the 30/12/2005 to the 06/01/2006 concurrent to MSLA map. b- Migration path in relation to weekly absolute dynamic topography (MADT) of an Argos-tracked leatherback turtle (FG05-2) nesting in French Guiana in July 2005. The fine line represents the turtle's track from 01/10/2005 to 24/02/2006 while the bold line represents the week from the 25/10/2005 to the 01/11/2005 (TRA 1) concurrent to MADT map. c- Migration path in relation to chlorophyll a surface concentration of an Argos-tracked leatherback turtle (GA06-1) nesting in Gabon in March 2006. The fine line represents the turtle's track from 04/03/2006 to 21/02/2007 while the bold line represents the period from the 01/06/2006 to the 30/06/2006 (TRA 13) concurrent to [Chla] map.

For two turtles (FG05-1 and FG05-3) no temporary residence areas were identified possibly due to the relatively short duration of their tracks (<4 months) and/or the low quality of the data towards the end of the tracks. For the 14 remaining turtles, TRAs were located both in the neritic (e.g. TRA7, 10, 21 **Figs. 1** **,** **2**) and the oceanic zone (e.g. TRA1, 11, 13; **Figs. 1** **,** **2**) and were characterised by a high diversity of oceanographic conditions. Amongst the neritic TRAs, one (TRA21) was located in the estuary of the Rio de la Plata characterised by a high chlorophyll *a* surface concentration whereas others (e.g. TRA2, 7, 10) were located on the edge of continental shelves with a steep slope. Amongst oceanic TRAs, two were located in highly dynamic areas characterised by important mesoscale eddy activity: the Gulf Stream (TRA11, **Fig 4a**) and the Brazil/Malvinas Confluence (TRA22), others were located in the Azores Current (TRA1), the Guinea Dome area (TRA3) and the South Equatorial Current (TRA12, 13, 16) characterised by oceanic fronts clearly highlighted in maps of absolute dynamic topography (MADT, **Fig. 4b**). All TRAs of Gabonese turtles were situated in the South Equatorial Current characterised by high chlorophyll *a* surface concentrations (**Fig. 4c**).

### From the nesting site to the first temporary residence area

**Figure 5:**
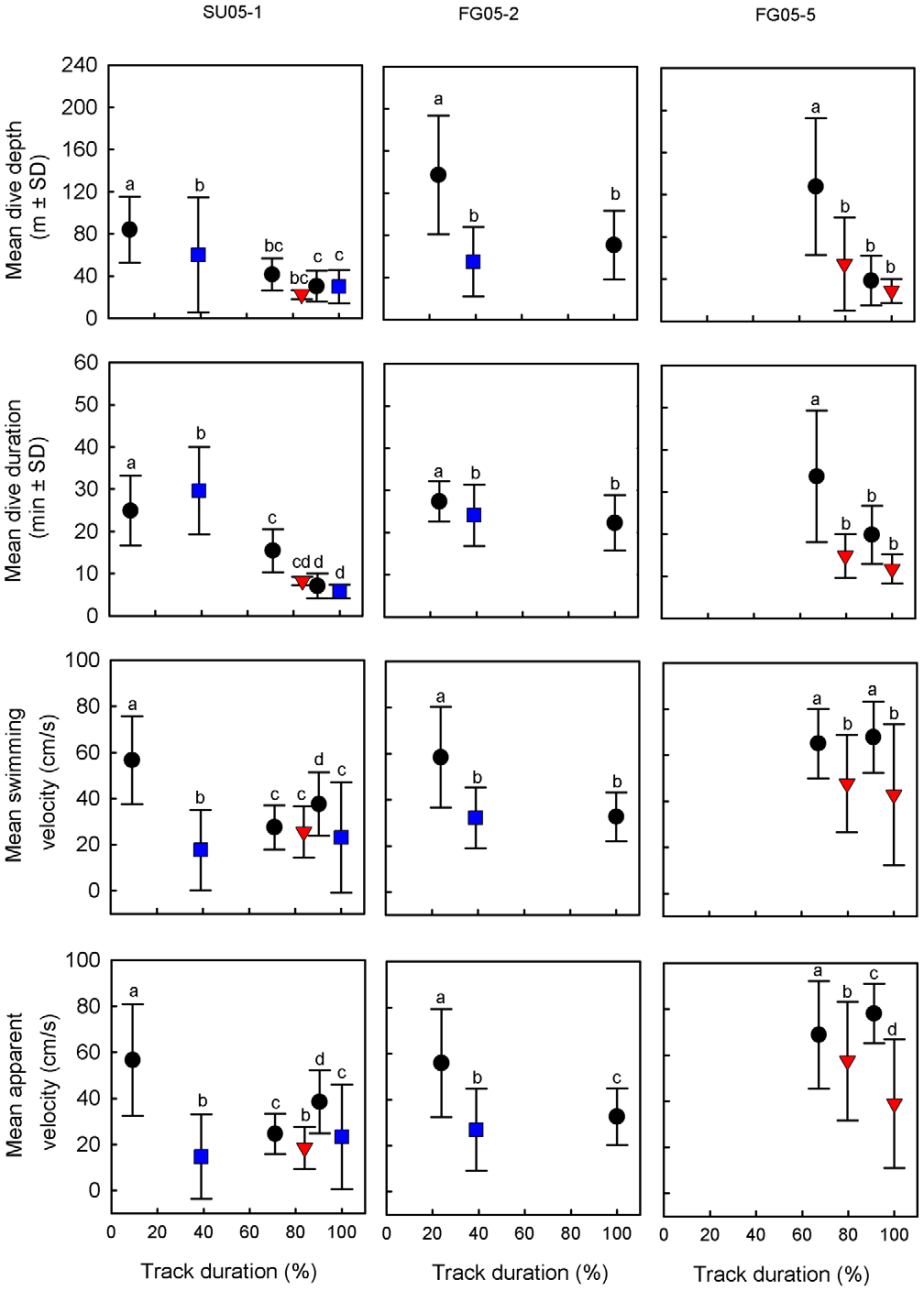
Variation in diving behaviour and velocities between areas. Diving behaviour and velocities in transit areas (filled dots), oceanic TRAs (filled crossed squares) and neritic TRAs (filled crossed triangles) for three Argos-tracked leatherback turtles nesting in Suriname (SU05-1) and French Guiana (FG05-2 and FG05-5) during their migrations in 2005. Differences between track sections were statistically tested using Kruskal-Wallis test followed by a post-hoc Bonferroni test. Different letters indicate significant (p<0.05) differences among areas. Values are expressed as mean ± SD.

All turtles satellite-tagged on their nesting beach reached their first TRA after 21 to 99 days of transit with a high mean swimming and apparent velocities (typically >45 cm.s^−1^, i.e. 39 km.day^−1^, except GA06-3, **Table S1,** **Fig. 5**) and a high mean straightness index of the motor and apparent paths (mean *D/L* typically >0.8). Turtles from Suriname/French Guiana and Panama performed long and deep dives (typically >20 min and >80 m respectively, **Table S1,** **Fig. 5**), although spending on average half of their time between 0–10 m deep (**Table S1**). Turtles from Gabon spent a lower percentage of time between 0–10 m deep compared to other turtles and performed shallower dives (**Table S1**).

### From transit areas to temporary residence areas

As turtles reached a TRA, there were marked changes in their vertical and/or horizontal behaviour depending on the type of habitat they exploited.

The passage from a neritic transit area to a neritic TRA (FG05-5, PA05-5, UR06-2, UR06-3) was associated with a decrease in swimming velocity (Kruskal-Wallis followed by a post-hoc Bonferroni test, p<0.05 in all cases, **Table S1**, **Fig. 5**) and in the mean straightness index for the motor path while dive parameters remained similar except for UR06-2 and UR06-3 for which dive depth decreased.

The passage from an oceanic transit area to a neritic TRA (FG05-5, SU05-1, PA05-4, PA05-5, UR05-1, UR06-2, UR06-3) was associated with a decrease in swimming velocity (p<0.05 in all cases, except SU05-1, **Table S1**, **Fig. 5**), in the mean straightness index for the motor path and in dive depth (p<0.05 in all cases, except SU05-1, **Table S1**, **Fig. 5**).

The passage from an oceanic transit area to an oceanic TRA (FG05-2, FG05-4, SU05-1, PA05-2, GA06-1, GA06-2, GA06-3, UR05-1, UR06-3) was associated with a decrease in swimming velocity (p<0.05 in all cases, except UR06-3, **Table S1**, **Fig. 5**) while the change in straightness index was more variable. Dive depth decreased for all turtles when they reached their first oceanic TRA (p<0.05 in all cases, **Table S1**, **Fig. 5**) except Gabonese turtles for which dive depth increased. However, when turtles reached subsequent oceanic TRAs their diving patterns did not change.

The passage from a neritic transit area to an oceanic TRA occurred only once (PA05-4) and was associated with an increase in dive duration (**Table S1**).

### Within neritic temporary residence areas

Within neritic TRAs, the mean swimming and apparent velocities were typically low (<45 cm.s^−1^, i.e. 39 km.day^−1^, **Table S1,** **Fig. 5**) with a lower straightness index along the motor and apparent paths than before reaching the TRA (mean *D/L* typically <0.8). Within neritic TRAs, turtles spent a majority of their time in the upper water column with more than 40% of their time spent between 0–10 m (up to 69% for SU05-1, **Table S1**) while dives were typically shallow (<50 m) and short (<20 min, **Table S1,** **Fig. 5**). Turtles PA05-4 and PA05-5 as they mostly remained along the continental slope of the Gulf of Mexico performed deeper (between 60 and 140 m) and longer (typically >20 min) dives. Compared to transit areas, the diving effort in term of total number of dives per hour increased regardless the initial domain (neritic or oceanic) they came from.

### Within oceanic temporary residence areas

Within oceanic TRAs, mean swimming and apparent velocities were highly variable among individuals depending on the actual oceanic dynamics assessed through current velocity (**Table S1,** **Fig. 5**). Accordingly turtles showed variable spatial structure of their path (i.e. path straightness) while remaining within an oceanic TRA: (1) in fast-current TRAs such as the Brazil/Malvinas Confluence and the Gulf Stream, turtles UR06-3 and PA05-2 had relatively fast swimming and apparent velocities (typically >45 cm.s^−1^, i.e. 39 km.day^−1^,) but a relatively lower straightness index for both the motor and apparent paths (typically <0.8). (2) Yet, in similar fast-current oceanic TRAs such as the Loop Current, turtle PA05-4 showed a high straightness index for its motor path, a high swimming velocity opposite to the main current resulting in a slow apparent velocity and a low straightness index for the apparent path. (3) Conversely, in low-current oceanic TRAs, such as the South Equatorial Tropical Gyre, turtle UR05-1 showed low swimming and apparent velocities (typically <30 cm.s^−1^, i.e. 26 km.day^−1^) but a high straightness index for both motor and apparent paths (typically >0.8) whereas turtles SU05-1, FG05-2 and FG05-4 showed a low straightness index for the motor path with similar low swimming and apparent velocities (typically <35 cm.s^−1^, i.e. 30 km.day^−1^). (4) Finally, all three Gabonese turtles showed low apparent velocities (typically <30 cm.s^−1^, i.e. 26 km.day^−1^) in the South Equatorial Tropical Gyre with either low (GA06-1) or high (GA06-2 and GA06-3) straightness index for the apparent paths.

Within oceanic TRAs, mean dive depth and mean dive duration were typically between 50–80 m (except UR06-3, **Table S1,** **Fig. 5**) and >20 min (except PA05-2 and UR06-3, **Table S1,** **Fig. 5**), respectively, with a high percentage of time spent between 0–10 m deep (typically >50%, except PA05-4 and GA06-2, **Table S1**).

## Discussion

For the last ten years, many studies have investigated in detail the diving behaviour and movements of leatherback turtles during their migration cycle in the Atlantic Ocean [19]–[21], [24]–[35]. For instance, in the North Atlantic, Ferraroli et al. [19] and Hays et al. [29] tracked females from their nesting sites in French Guiana and Grenada, respectively, while James et al. [31], [32] tracked male and female leatherback turtles from an important foraging site in Nova Scotia. Evans et al. [26] described the migration patterns in the Gulf of Mexico of females nesting in Panama whereas in the South Atlantic, the recent study of López-Mendilaharsu et al. [20] focused on the behaviour of turtles captured in the Southwestern Atlantic Ocean. Yet to date, only one study concurrently investigated the migratory behaviour of leatherback turtles from both nesting and foraging sites in the North Atlantic basin [27]. The present study similarly brings together individual tracks but from three major nesting sites and one recently identified foraging area over the North and South Atlantic Ocean to identify temporary residence areas and associated environmental determinants. As such this study provides a new point of view on leatherback migration patterns and complements previously published works.

### Atlantic migratory paths and TRAs

By monitoring 16 leatherback turtles from three nesting sites and one foraging area over the Atlantic ocean, this study clearly illustrates that the general dispersal patterns and TRAs used by the turtles may vary among individuals of a same nesting population and among populations. For instance females tracked from the nesting sites in French Guiana and Suriname only dispersed through the North Atlantic basin heading broadly northwest, northeast, or east (this study and [19], [27]) whereas two of the three females tracked from their nesting beach in Panama dispersed in the Gulf of Mexico and the third one reached the Gulf Stream area (this study and [26]). To date, no satellite-tracked females from the Caribbean, French Guiana or Suriname nesting populations have ever entered the Gulf of Mexico or travelled south to the South Atlantic. In the Southern hemisphere, all three females tracked from Gabon dispersed through the South Atlantic basin mainly remaining within the South Equatorial Current while the turtles captured in coastal and oceanic waters off South America remained in the Southwestern Atlantic (this study and [20]). So within nesting populations, there is a tendency for migratory paths to be broadly similar (i.e. remaining within the same ocean body such as North Atlantic or Gulf of Mexico) but with large variation existing between the extreme paths taken (e.g. FG05-5 and FG05-3). Yet, there is a much greater variability of migratory paths between populations.

We identified 22 TRAs distributed throughout the Atlantic Ocean, 9 in the neritic domain and 13 in the oceanic domain. This corroborates previous studies suggesting that leatherback turtles are both oceanic and neritic foragers [20], [25], [40]. As a consequence, these TRAs did not share a common oceanographic determinant but on the contrary were associated with mesoscale surface oceanographic features of different types (i.e. altimetric features and/or surface chlorophyll *a* concentration). Several TRAs were located in distinct oceanic frontal zones and eddies. The importance of oceanographic fronts to this species, but also to marine birds and mammals (review in [41]) has already been described [19], [24], [34], [42]. Other TRAs were located in estuaries and along coastal shelf breaks that constitute sharp water density discontinuities where biomass concentrates, including gelatinous zooplankton, the leatherback prey [43]–[45]. Slope waters seem indeed of important use for leatherback turtles. For instance, turtles PA05-4 and PA05-5 spent most of their time along the continental slope of the Gulf of Mexico, maybe foraging on gelatinous zooplankton aggregated along the shelf-break front [43]. All TRAs used by the turtles have been previously described as productive areas: e.g. the Mauritania upwelling [46], the Gulf of Mexico [47], the Gulf Stream [48], the Brazil/Malvinas Confluence [49], and the estuary of Rio de la Plata [50], [51] suggesting that TRAs may indeed be associated with foraging. In addition, several TRAs identified in this study closely match the high-foraging success areas previously identified for leatherback turtles during their pluri-annual migration in the North Atlantic [27]. Interestingly, individuals from a same nesting area may show contrasting patterns in habitat use such as PA05-5 only exploiting oceanic TRAs and PA05-2 only neritic ones. Migratory paths and habitat use patterns in the leatherback turtle thus are both characterized by high intra- and inter-population variation.

### Vertical and horizontal behaviours within TRAs

Despite highly variable oceanographic conditions among TRAs, turtles interestingly rather exhibited relatively similar horizontal and vertical behaviours when in TRAs. First, when taking into account the influence of surface currents on the horizontal behaviour of the animals, it appears that, in general, turtles slowed down their swimming velocity as they reached TRAs and exhibited highly sinuous motor and apparent paths. This may be associated with area-restricted searching (ARS) patterns that other marine predators display when foraging [52]–[54]. However, in certain cases this general behaviour was shaped by local current conditions. This was revealed by the method used in this study which assesses the contribution of both the animal and the environmental cues to the way an animal remains in TRAs. For instance, within zones of high mesoscale activity (presence of many eddies) turtles rather increased their swimming velocities while performing sinuous movements to remain in the productive patch (e.g. turtles UR06-3 and PA05-2). An interesting case is the turtle PA05-4 that remained at the edge of the Loop Current for several months showing a highly sinuous apparent path and a low corresponding velocity but a straight motor path and high swimming velocity. This suggests that during several months, the turtle headed in a direction opposed to the Loop Current while she apparently remained in a restricted area looping within the flow. This behaviour might be an original strategy by which turtles feed at counter-current. Indeed, swimming at counter-current allows an animal to prospect water mass and thus potentially a prey patch without moving with respect to the sea bottom. Such behaviour may provide some benefits, as, for example, in terms of orientation by limiting extensive drifts throughout the oceanic basin, or in terms of foraging by maintaining the animal in an area where surface resources availability may be driven by deep, bathymetric-mediated, oceanic processes. This behaviour has been previously suggested for a leatherback turtle foraging in the Azores Current [37]. Different horizontal tactics seem thus to be used by the turtles to remain in a productive patch according to local oceanographic conditions. This highlights the necessity to cautiously interpret horizontal movement patterns in marine predators in relation to contemporaneous environmental dynamics [22], [37]. Novel tracking technologies such as fastloc® GPS loggers by improving accuracy in tracking marine species [55] may help resolving the underlying patterns of movement in great details and allow a better understanding of relationships with environmental parameters.

Shallow diving behaviour was observed in all TRAs at all latitudes in a relatively homogenous way among individuals. In oceanic TRAs, dives were longer (>20 min) than in neritic TRAs and mainly concentrated in the epipelagic layer (50–80 m). This suggests that the diving behaviour was shaped by local prey distribution and density, as described for other marine vertebrates (e.g., [56], [57]). Periods of very short shallow dives and high use of surface waters have previously been reported for leatherback turtles foraging at high latitude [24], [28], [33] where gelatinous plankton is available at shallow depths [58], [59]. Similar pattern was described in basking sharks (*Cetorhinus maximus*) foraging on continental shelves [52], [57]. Higher variability in diving behaviour was observed in oceanic TRAs. Such variability in oceanic areas has also been observed in other marine species, particularly sea birds [60] and is likely driven by the stochastic nature of the oceanic environment resulting in less predictable and patchily distributed prey. This suggests that in neritic and geographically well-delimited TRAs, such as the Rio de la Plata estuary, where turtles exhibit relatively consistent diving patterns, spatio-temporal fishing regulations to mitigate bycatch may be more easily designed than in oceanic TRAs.

### TRA fidelity and hatchling drift hypothesis

On one occasion, two individuals, one from the Southeast Atlantic and one from the Southwest Atlantic, stayed in the same TRA suggesting a potential connection between turtles from both sides of the South Atlantic. Leatherback turtles flipper-tagged on the beaches of Gabon have indeed previously been recovered in the waters of Argentina and Brazil [18] suggesting that turtles captured in international waters of the Southwest Atlantic likely belong to the West African nesting populations. Among the 16 turtles tracked in this study, several of them showed strong fidelity to TRAs (**Fig. 2**). Fidelity to a specific area has already been described in leatherback turtles foraging in Nova Scotia and in the Rio de la Plata estuary [20], [32] but also in other sea turtle species [61]. Such behaviour is counterintuitive considering the high variability in post-breeding migration destinations observed among turtles of a given nesting population or among nesting populations. Yet, both may be linked to initial hatchling drift patterns [16], [17]. The possible drift scenarios of hatchling turtles dispersing from their nesting sites may be inferred by looking at passive drifter trajectories. Here most of the individual dispersal patterns observed in the North Atlantic, the South Atlantic and the Gulf of Mexico showed interesting similarities with the trajectories of some satellite-tracked drifters (**Figs 1** **,** **3**), although such data should be taken with caution as they were collected at different periods. In addition, most of the TRAs used by adult turtles during their post-breeding migrations were located along the drifter trajectories corroborating the “hatchling drift scenario” hypothesis [16]. Indeed, it has been suggested that hatchling turtles may imprint on several possible future and predictable foraging sites during the years when they are passively carried by ocean currents. Then, as adults they may make the decision to go to the preferred site(s) based on that initial experience and may follow the same routes [16], [17]. Clearly, not all hatchling drift patterns generate possible scenarios for adult migration because of differential mortality rate between oceanographic areas (Gaspar et al. submitted). In addition, not all adult migration patterns match a hatchling drift scenario. For instance, in this study, some females left French Guiana and crossed the North Atlantic Gyre in a southwest-northeast direction heading towards the Azores. In this area, ocean currents are very weak and such trajectory could not occur by passive drift. Many other drifter trajectories end up however around the Azores which indeed represent a TRA used by many turtles (this study and [24], [27]). This suggests that adult leatherback turtles may return to specific sites previously explored in their early lives without, however, always following the same routes as hatchlings but rather use shortcuts.

### Conclusion

Identification of habitat use and associated diving behaviour is the first step for effective conservation of marine vertebrates. In this study, 22 temporary residence areas that may correspond to foraging areas have been identified in contrasted oceanographic environments ranging from neritic to oceanic domains for 16 Atlantic leatherback turtles. The observed migratory paths and TRAs distributions appear to be related to multiple oceanographic conditions, and may be linked with initial hatchling drift scenarios [16]. This study thus highlights the importance but also the difficulty of implementing spatio-temporal fishing regulations over a large geographical scale and suggests that modification of fishing gears and fishing behaviours might be more efficient to protect such highly migratory species. Despite the sample size and diversity of study sites used in this study, it also appears that a larger multi-year dataset (at least >100 individuals) is needed through international collaborative efforts for providing general recommendations in terms of conservation of this critically-endangered species.

## Supporting Information

Table S1 Summary of diving behaviour, swimming/apparent/current velocities and time spent in transit area/temporary residence area (TRA)/inter-TRA in oceanic (O) or neritic (N) domains in 16 Argos tracked leatherback turtles during their migration between 2005 and 2008 (see Fig. 1). Transit areas correspond to the time turtles spent from their nesting beach to their first TRA. TRAs correspond to 1° * 1° areas where turtles spent more than 90 hours. Inter- TRAs correspond to the time turtles spent between two TRAs (see M&M for details). * for PA05-2, the 35 days at the end of the track were not taken into account due to the very few numbers of locations obtained during this period. Differences between areas were statistically tested using Kruskal-Wallis test followed by a post-hoc Bonferroni test. Different letters indicate significant (p<0.05) differences among areas. Values are expressed as mean ± SD.
(0.12 MB DOC)
