# Supplementary material for: Atlantic Leatherback Migratory Paths and Temporary Residence Areas
Source: PLoS One. 2010 Nov 9;5(11):e13908. doi: 10.1371/journal.pone.0013908 (PMC2976686; doi:10.1371/journal.pone.0013908)
Supplement: Table S1 — Summary of diving behaviour, swimming/apparent/current velocities and time spent in transit area/temporary residence area (TRA)/inter-TRA in oceanic (O) or neritic (N) domains in 16 Argos tracked leatherback turtles during their migration between 2005 and 2008 (see Fig. 1). Transit areas correspond to the time turtles spent from their nesting beach to their first TRA. TRAs correspond to 1° * 1° areas where turtles spent more than 90 hours. Inter- TRAs correspond to the time turtles spent between two TRAs (see M&M for details). * for PA05-2, the 35 days at the end of the track were not taken into account due to the very few numbers of locations obtained during this period. Differences between areas were statistically tested using Kruskal-Wallis test followed by a post-hoc Bonferroni test. Different letters indicate significant (p<0.05) differences among areas. Values are expressed as mean ± SD. (0.12 MB DOC) [file pone.0013908.s001.doc]

| Turtles | Type of area | Dive depth (m) | Dive duration (min) | Time spent between 0-10 m (%) | Swimming velocity (cm.s^-1^) | Apparent velocity  (cm.s^-1^) | Current velocity (cm.s^-1^) | Time in days (%) |
| --- | --- | --- | --- | --- | --- | --- | --- | --- |
|  |  |  |  |  |  |  |  |  |
| FG05-2 | Transit | 137.2 ± 56.2 ^a^ | 27.4 ± 4.8 ^a^ | 50.5 ± 27.9 ^a^ | 58.5 ± 21.9 ^a^ | 55.9 ± 23.5 ^a^ | 11.2 ± 8.6 ^a^ | 97.9 (23.9) |
|  | **TRA-O** | **54.8 ± 32.9 ^b^** | **24.1 ± 7.3 ^b^** | **61.9 ± 27.3 ^a^** | **32.2 ± 13.2 ^b^** | **26.9 ± 17.8 ^b^** | **12.0 ± 4.9 ^a^** | **62.0 (15.1)** |
|  | Inter-TRA O | 70.7 ± 32.4 ^b^ | 22.3 ± 6.5 ^b^ | 60.7 ± 27.0 ^a^ | 32.6 ± 10.6 ^b^ | 32.6 ± 12.2 ^c^ | 10.9 ± 6.0 ^a^ | 250.5 (61.0) |
|  |  |  |  |  |  |  |  |  |
| FG05-4 | Transit | 114.0 ± 41.7 ^a^ | 31.8 ± 9.7 ^a^ | 53.4 ± 28.7 ^a^ | 67.9 ± 13.1 ^a^ | 69.0 ± 14.8 ^a^ | 12.3 ± 12.1 ^a^ | 86.0 (83.6) |
|  | **TRA-O** | **58.3 ± 18.9 ^b^** | **31.1 ± 6.1 ^a^** | **78.2 ± 16.9 ^b^** | **33.2 ± 25.6 ^b^** | **27.4 ± 31.0 ^b^** | **12.3 ± 5.6 ^a^** | **16.9 (16.4)** |
|  |  |  |  |  |  |  |  |  |
| FG05-5 | Transit | 127.6 ± 64.8 ^a^ | 33.7 ± 15.6 ^a^ | 53.5 ± 33.5 ^a^ | 65.1 ± 15.1 ^a^ | 68.8 ± 23.4 ^a^ | 17.0 ± 19.3 ^a^ | 75.6 (66.6) |
|  | **TRA-N** | **46.8 ± 39.0 ^b^** | **14.0 ± 4.9 ^b^** | **52.0 ± 24.0 ^a^** | **45.7 ± 25.5 ^b^** | **49.8 ± 28.2 ^b^** | **15.9 ± 7.3 ^a^** | **23.9 (21.0)** |
|  | Inter-TRA N | 38.5 ± 23.5 ^b^ | 19.9 ± 6.9 ^b^ | 64.1 ± 25.0 ^a^ | 67.8 ± 15.4 ^a^ | 78.1 ± 12.9 ^c^ | 15.1 ± 5.7 ^a^ | 14.0 (12.3) |
|  |  |  |  |  |  |  |  |  |
| SU05-1 | Transit | 84.2 ± 31.5 ^a^ | 24.9 ± 8.3 ^a^ | 43.2 ± 27.7 ^a^ | 56.6 ± 19.1 ^a^ | 56.6 ± 24.2 ^a^ | 15.0 ± 13.8 ^a^ | 65.9 ( 9.2) |
|  | **TRA-O** | **50.1 ± 47.4 ^b^** | **21.6 ± 14.2 ^a^** | **56.8 ± 28.3 ^b^** | **24.2 ± 18.7 ^b^** | **23.5 ± 18.4 ^b^** | **8.3 ± 4.9 ^b^** | **381.9 (53.4)** |
|  | Inter-TRA O | 37.9 ± 15.8 ^b^ | 12.7 ± 6.0 ^b^ | 58.9 ± 26.0 ^b^ | 29.2 ± 13.3 ^c^ | 29.1 ± 13.5 ^c^ | 8.3 ± 6.2 ^b^ | 247.6 (34.6) |
|  | **TRA-N** | **22.7 ± 4.3 ^b^** | **8.3 ± 0.9 ^b^** | **68.9 ± 14.2 ^a b^** | **44.1 ± 26.2 ^d^** | **36.2 ± 22.4 ^b c^** | **26.2 ± 2.4 ^c^** | **20.0 ( 2.8)** |
|  |  |  |  |  |  |  |  |  |
| PA05-2* | Transit | 105.6 ± 39.5 ^a^ | 25.9 ± 7.3 ^a^ | 76.6 ± 24.2 ^a^ | 68.8 ± 20.0 ^a^ | 64.5 ± 21.5 ^a^ | 21.1 ± 15.1 ^a^ | 119.0 (19.9) |
|  | **TRA-O** | **58.5 ± 38.5 ^b^** | **14.9 ± 5.9 ^b^** | **63.4 ± 25.7 ^a^** | **42.8 ± 24.8 ^b^** | **51.7 ± 33.9 ^b^** | **49.3 ± 25.4 ^b^** | **145.0 (24.3)** |
|  | Inter-TRA O | 42.6 ± 28.8 ^c^ | 17.6 ± 8.4 ^c^ | 64.9 ± 23.6 ^a^ | 53.4 ± 31.2 ^c^ | 60.4 ± 50.8 ^a^ | 38.6 ± 33.4 ^c^ | 333.0 (55.8) |
|  |  |  |  |  |  |  |  |  |
| PA05-4 | Transit | 113.3 ± 56.6 ^a^ | 24.7 ± 7.6 ^a^ | 42.5 ± 21.9 ^a b^ | 57.0 ± 16.3 ^a^ | 73.1 ± 18.0 ^a^ | 26.2 ± 16.8 ^a^ | 36.9 (10.2) |
|  | **TRA-N** | **61.9 ± 33.0 ^b^** | **21.5 ± 12.9 ^a^** | **48.2 ± 22.5 ^a^** | **39.6 ± 19.8 ^b^** | **37.9 ± 19.9 ^b^** | **12.6 ± 12.2 ^b^** | **88.0 (24.3)** |
|  | Inter-TRA N | 59.8 ± 53.3 ^b^ | 25.0 ± 13.4 ^a^ | 67.9 ± 29.0 ^c^ | 32.4 ± 13.9 ^b c^ | 32.1 ± 33.0 ^b^ | 26.5 ± 20.0 ^a^ | 112.5 (31.0) |
|  | **TRA-O** | **64.1 ± 43.9 ^b^** | **34.0 ± 12.7 ^b^** | **36.1 ± 23.6 ^b^** | **45.8 ± 25.8 ^c^** | **34.7 ± 29.3 ^b^** | **44.2 ± 16.9 ^c^** | **125.0 (34.5)** |
|  |  |  |  |  |  |  |  |  |
| PA05-5 | Transit | 109.5 ± 72.7 ^a^ | 22.4 ± 8.8 ^a^ | 59.9 ± 22.4 ^a^ | 71.2 ± 16.9 ^a^ | 97.3 ± 33.4 ^a^ | 33.4 ± 28.6 ^a^ | 24.0 ( 7.4) |
|  | **TRA-N** | **76.2 ± 41.5 ^b^** | **28.8 ± 14.8 ^a b^** | **44.1 ± 25.4 ^b^** | **32.0 ± 18.1 ^b^** | **32.4 ± 19.2 ^b^** | **18.3 ± 12.8 ^b^** | **225.3 (69.4)** |
|  | Inter-TRA N | 114.7 ± 39.5 ^a b^ | 33.5 ± 5.1 ^a b^ | 35.9 ± 24.3 ^a b^ | 59.1 ± 29.5 ^c^ | 64.4 ± 26.8 ^c^ | 31.2 ± 24.3 ^a^ | 24.4 ( 7.5) |
|  | Inter-TRA O | 96.0 ± 34.1 ^a^ | 29.5 ± 9.2 ^b^ | 35.9 ± 23.5 ^b^ | 39.4 ± 21.1 ^d^ | 49.4 ± 22.5 ^d^ | 24.3 ± 11.7 ^c^ | 51.0 (15.7) |
|  |  |  |  |  |  |  |  |  |
| GA06-1 | Transit | 52.5 ± 13.8 ^a^ | 19.8 ± 6.3 ^a^ | 62.6 ± 28.0 ^a^ | - | 52.0 ± 15.3 ^a^ | - | 88.6 (16.6) |
|  | **TRA-O** | **68.0 ± 42.5 ^b^** | **26.4 ± 10.6 ^b^** | **63.4 ± 24.1 ^a^** | **-** | **21.2 ± 12.6 ^b^** | **-** | **364.8 (68.5)** |
|  | Inter-TRA O | 71.5 ± 39.8 ^a b^ | 29.9 ± 9.9 ^b^ | 63.5 ± 27.4 ^a^ | - | 45.9 ± 15.5 ^c^ | - | 79.5 (14.9) |
|  |  |  |  |  |  |  |  |  |
| GA06-2 | Transit | 59.4 ± 17.8 ^a^ | 24.6 ± 8.4 ^a^ | 31.3 ± 13.0 ^a^ | - | 57.6 ± 24.0 ^a^ | - | 20.8 (19.0) |
|  | **TRA-O** | **63.2 ± 18.4 ^a^** | **31.0 ± 6.9 ^a^** | **38.8 ± 29.5 ^a b^** | **-** | **20.8 ± 23.2 ^b^** | **-** | **63.5 (58.1)** |
|  | Inter-TRA O | 67.8 ± 1.0 ^a^ | 31.3 ± 3.5 ^a^ | 65.3 ± 41.7 ^b^ | - | 44.3 ± 12.9 ^c^ | - | 25.0 (22.9) |
|  |  |  |  |  |  |  |  |  |
| GA06-3 | Transit | 46.6 ± 12.4 ^a^ | 18.0 ± 5.5 ^a^ | 44.6 ± 23.7 ^a^ | - | 36.8 ± 13.6 ^a^ | - | 74.8 (25.0) |
|  | **TRA-O** | **55.5 ± 21.5 ^b^** | **20.6 ± 6.4 ^a b^** | **56.9 ± 22.2 ^a^** | **-** | **27.0 ± 16.2 ^b^** | **-** | **55.0 (18.4)** |
|  | Inter-TRA O | 60.7 ± 18.6 ^b^ | 24.4 ± 6.2 ^b^ | 56.4 ± 23.4 ^a^ | - | 30.5 ± 20.0 ^b^ | - | 169.5 (56.6) |
|  |  |  |  |  |  |  |  |  |
| UR05-1 | **TRA-O** | **76.7 ± 16.3 ^a^** | **20.3 ± 8.5 ^a^** | **46.6 ± 17.3 ^a^** | **28.3 ± 11.4 ^a^** | **24.3 ± 13.2 ^a^** | **8.2 ± 4.1 ^a^** | **64.5 (20.6)** |
|  | Inter-TRA O | 73.7 ± 30.5 ^a^ | 29.5 ± 7.3 ^b^ | 46.0 ± 26.9 ^a^ | 32.5 ± 13.9 ^b^ | 34.1 ± 14.4 ^b^ | 10.3 ± 7.2 ^b^ | 239.0 (76.1) |
|  | **TRA-N** | **25.3 ± 5.7 ^b^** | **13.2 ± 1.4 ^a^** | **52.9 ± 1.3 ^a^** | **24.7 ± 19.3 ^a^** | **23.2 ± 19.7 ^a^** | **24.1 ± 8.7 ^c^** | **10.5 ( 3.3)** |
|  |  |  |  |  |  |  |  |  |
| UR06-1 | **TRA-N** | **42.2 ± 20.8** | **20.3 ± 9.2** | **68.4 ± 22.9** | **26.3 ± 16.3** | **29.2 ± 20.9** | **19.3 ± 10.1** | **339.8 (100.0)** |
|  |  |  |  |  |  |  |  |  |
| UR06-2 | Inter-TRA O | 58.3 ± 24.0^a^ | 22.0 ± 5.5 ^b^ | 50.3 ± 17.1 ^a^ | 58.3 ± 12.3 ^a^ | 48.9 ± 19.0 ^a^ | 19.3 ± 8.8 ^a^ | 19.4 ( 8.2) |
|  | **TRA-N** | **24.1 ± 19.7^b^** | **14.1 ± 11.9 ^a^** | **57.5 ± 23.6 ^a^** | **29.2 ± 18.1 ^b^** | **20.5 ± 14.2 ^b^** | **20.4 ± 14.3 ^a^** | **166.9 (70.3)** |
|  | Inter-TRA N | 33.2 ± 17.8^c^ | 17.4 ± 8.4 ^a^ ^b^ | 59.6 ± 22.9 ^a^ | 56.6 ± 22.0 ^a^ | 62.1 ± 25.6 ^c^ | 20.9 ± 9.4 ^a^ | 51.0 (21.5) |
|  |  |  |  |  |  |  |  |  |
| UR06-3 | **TRA-O** | **36.9 ± 24.0 ^a^** | **14.7 ± 5.1 ^a^** | **77.2 ± 20.7 ^a^** | **52.3 ± 22.8 ^a^** | **48.4 ± 28.0 ^a^** | **46.3 ± 20.9 ^a^** | **52.0 ( 8.3)** |
|  | Inter-TRA O | 36.2 ± 19.9 ^a^ | 18.2 ± 8.3 ^a^ | 72.1 ± 28.4 ^a^ | 45.9 ± 24.8 ^a,c^ | 55.3 ± 34.0 ^b^ | 39.8 ± 21.2 ^b^ | 61.6 ( 9.8) |
|  | **TRA-N** | **16.4 ± 10.1 ^b^** | **16.1 ± 11.6 ^a^** | **50.3 ± 26.7 ^b^** | **35.1 ± 16.3 ^b^** | **24.0 ± 16.6 ^c^** | **25.4 ± 15.3 ^c^** | **262.3 (41.7)** |
|  | Inter-TRA N | 32.0 ± 13.3 ^a^ | 25.8 ± 14.7 ^b^ | 59.9 ± 29.5 ^c^ | 40.6 ± 20.6 ^c^ | 48.5 ± 29.3 ^a,b^ | 18.6 ± 16.7 ^d^ | 253.7 (40.3) |

Table S1. Summary of diving behaviour, swimming/apparent/current velocities and time spent in transit area / temporary residence area (TRA) / inter-TRA in oceanic (O) or neritic (N) domains in 16 Argos tracked leatherback turtles during their migration between 2005 and 2008 (see Fig. 1). Transit areas correspond to the time turtles spent from their nesting beach to their first TRA. TRAs correspond to 1° * 1° areas where turtles spent more than 90 hours. Inter- TRAs correspond to the time turtles spent between two TRAs (see M&M for details). * for PA05-2, the 35 days at the end of the track were not taken into account due to the very few numbers of locations obtained during this period. Differences between areas were statistically tested using Kruskal-Wallis test followed by a post-hoc Bonferroni test. Different letters indicate significant (p < 0.05) differences among areas. Values are expressed as mean ± SD.
